# Elevated peripheral levels of receptor-interacting protein kinase 1 (RIPK1) and IL-8 as biomarkers of human amyotrophic lateral sclerosis

**DOI:** 10.1038/s41392-023-01713-z

**Published:** 2023-12-13

**Authors:** Jun Wei, Min Li, Zhi Ye, Xinqian Hu, Xiaoyan He, Jia Wang, Gaofeng Chen, Chengyu Zou, Daichao Xu, Hongbing Zhang, Junying Yuan, Yunhong Zha

**Affiliations:** 1 Institute of Neural Regeneration and Repair and Department of Neurology, The First College of Clinical Medical Science, Yichang Central Hospital, College of Basic Medical Science, China Three Gorges University, Hubei Province Clinical Medical Research Center for Rare Diseases of Nervous System, Yichang, 443000 China; 2 Interdisciplinary Research Center on Biology and Chemistry, Shanghai Institute of Organic Chemistry, Chinese Academy of Sciences, Shanghai Key Laboratory of Aging Studies, Shanghai, 201210 China; 3 State Key Laboratory of Common Mechanism Research for Major Diseases, Haihe Laboratory of Cell Ecosystem, Department of Physiology, Institute of Basic Medical Sciences and School of Basic Medicine, Chinese Academy of Medical Sciences and Peking Union Medical College, Beijing, China

**Keywords:** Clinical trials, Target validation, Neurological disorders

## Abstract

Amyotrophic lateral sclerosis (ALS) is a devastating fatal neurodegenerative disease with no cure. Receptor-interacting protein kinase 1 (RIPK1) has been proposed to mediate pathogenesis of ALS. Primidone has been identified as an old drug that can also inhibit RIPK1 kinase. We conducted a drug-repurposing biomarker study of primidone as a RIPK1 inhibitor using SOD1^G93A^ mice and ALS patients. SOD1^G93A^ mice treated with primidone showed significant delay of symptomatic onset and improved motor performance. One-hundred-sixty-two ALS participants dosed daily with primidone (62.5 mg) completed 24-week follow-up. A significant reduction was showed in serum levels of RIPK1 and IL-8, which were significantly higher in ALS patients than that of healthy controls (*P* < 0.0001). Serum RIPK1 levels were correlated positively with the severity of bulbar symptoms (*P* < 0.05). Our study suggests that serum levels of RIPK1 and IL-8 in peripheral can be used as clinical biomarkers for the activation of RIPK1 in central nervous system in human ALS patients. Repurposing primidone may provide a promising therapeutic strategy for ALS. The effect of primidone for the treatment of other inflammatory diseases may also be considered, since the activation of RIPK1 has been implicated in mediating a variety of inflammatory diseases including COVID-19-associated cytokine release syndrome (CRS). (ChiCTR2200060149).

## Introduction

Amyotrophic lateral sclerosis (ALS) is a fatal neurodegenerative disease that usually results in death within 2–5 years after diagnosis. While majority of ALS cases are sporadic, 10–20% of the cases have familial recurrence (fALS) which may be linked to variants in one of the 40 genes identified.^1^ ALS is characterized by progressive loss of upper and lower motor neurons in motor cortex, brainstem and spinal cord leading to progressive muscle weakness. The onset of ALS in approximately one-third of patients occurs at the bulbar level (B-ALS) and the remaining two-third onset occurs at the spinal level with weakness and atrophy of the muscles in upper or lower limbs (L-ALS).^2^ Muscle dysfunction and atrophy in ALS progressively spread from one region to other regions. The B-ALS onset is characterized by bulbar symptoms such as dysarthria (slurred speech) and dysphagia (difficulties in swallowing), which can be associated with limb symptoms developed simultaneously or within 1–2 years.^3,4^ The development of bulbar symptoms also occur in almost all L-ALS at late stages. Bulbar paralysis is known to be closely associated with respiratory dysfunction, which eventually leads to respiratory failure, the cause of death in ALS. Effective therapies to halt or delay the pathological progression of ALS are an urgent unmet medical need.

Receptor-interacting protein kinase 1 (RIPK1) is a master mediator of multiple cell death pathways, including apoptosis and necroptosis, as well as inflammatory response.^5^ Activation of RIPK1 kinase in TNFR1 signaling pathway associated with complex I can promote the formation of downstream complex IIa for mediating RIPK1-dependent apoptosis (RDA) or with RIPK3 to form complex IIb for promoting necroptosis. Activation of RIPK1 has been observed in human ALS spinal cord samples.^6^ In studies involving mouse models of ALS, the activation of RIPK1 kinase has been demonstrated to promote neuroinflammation and progressive degeneration of motor neurons in the pathogenesis of ALS which can be inhibited by genetic and pharmacological inactivation of RIPK1.^6–8^ However, human clinical studies on developing a RIPK1 inhibitor for the treatment of ALS have been less than straight-forward. A RIPK1 inhibitor advanced into a Phase I human clinical trial for the treatment of ALS had to be terminated due to long-term dose-limiting toxity of the compound under investigation unrelated to the target.^9^ Recently, Sanofi and Denali advanced another RIPK1 inhibitor (SAR443820) into a Phase II multi-center, randomized, double-blind, placebo-controlled trial, named HIMALAYA trial, for the treatment of ALS (ClinicalTrials.gov ID: NCT05237284). New insights into the peripheral biomarkers of ALS patients may help to guide the ongoing ALS clinical trials.

We conducted a repurpositional biomarker study using an old drug, primidone which has been shown to also inhibit RIPK1,^10^ with animal ALS model and human ALS participants. Treatment with primidone delayed the onset and progression of motor deficits, histological pathology and body-weight loss of SOD^G93A^ mice, an ALS model, validating the effect of primidone to inhibit RIPK1 and rescue motor functions in vivo. In human ALS participants recruited in this study, we found that dosing with primidone reduced the serum levels of RIPK1 and IL-8, which were significantly higher in ALS patients than that of healthy individuals. The serum RIPK1 values in patients with bulbar onset (B-ALS) were higher than that in patients with limb onset (L-ALS). In addition, serum RIPK1 levels in patients with ALS are positively correlated with the severity of bulbar symptoms. These data demonstrate the value of RIPK1 and IL-8 in serum as biomarkers in clinical studies developing RIPK1 inhibitor for the treatment of ALS.

## Results

### Primidone mitigates ALS-like development of SOD1^G93A^ mice

As a neuronal voltage-gated sodium channel blocker, primidone has been used for the treatment of epilepsy, essential tremor, and psychiatric disorders.^11^ We performed in vitro kinase assays using Flag-tagged murine full-length RIPK1 using p-S166 RIPK1 as a biomarker for the activation of RIPK1.^12^ ~50% reduction RIPK1 kinase activity was achieved in the presense of 0.1–1 µM primidone and complete inhibition of RIPK1 kinase activity was observed with concentrations of primidone greater than or equal to 10 µM (Supplementary Fig. 1).

**Fig. 1:**
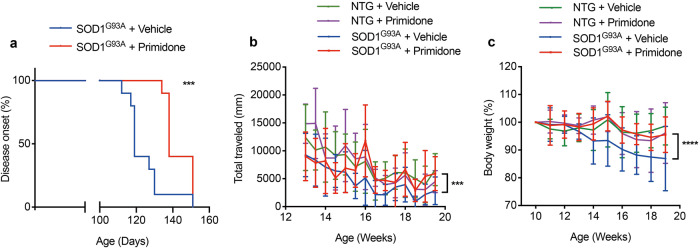
Primidone treatment mitigates ALS-like disease development in SOD1^G93A^ mice. **a** The effect of primidone dosing (primidone or vehicle treated from 10 weeks of age) on the onset of motor dysfunction in SOD1^G93A^ mice. **b** All mice were weighed once a week from 10 weeks to 19 weeks of age. NTG: non-transgenic mice. **c** All mice were tested mobility in open field test from 13 weeks to 20 weeks of age. Statistical analysis of onset was conducted with Log-rank (Mantel-Cox) test, ***P < 0.001. Data are expressed as mean ± SD, *n* = 10 mice/group, ***P < 0.001, ****P < 0.0001

To investigate the effect of primidone on ALS in vivo, we first studied the impact of primidone on SOD1^G93A^ mice, an animal model of ALS. SOD1^G93A^ mice develop age-dependent neurodegeneration and behavior dysfunction with resemblance to the clinic features in human ALS.^13^ Primidone (25 mg/kg/day) or vehicle alone was dosed orally to SOD1^G93A^ mice from 10 weeks of age to the end-stage. Compared to that of vehicle-treated SOD1^G93A^ mice, primidone-treatment delayed the symptomatic onset (*P* < 0.001), reduced the body weight loss (*P* < 0.0001) and improved the motor performance (*P* < 0.001) (Fig. 1a–c). No significant difference in the survival times was observed between vehicle-treatment and primidone-treatment SOD1^G93A^ mice (Supplementary Fig. 2). These data suggest that primidone dosed at 25 mg/kg/day can delay the onset of motor deficit, improve neurological scores and reduce the loss of body weight in SOD1^G93A^ transgenic mice, but may not affect the life span of this ALS model.

**Fig. 2:**
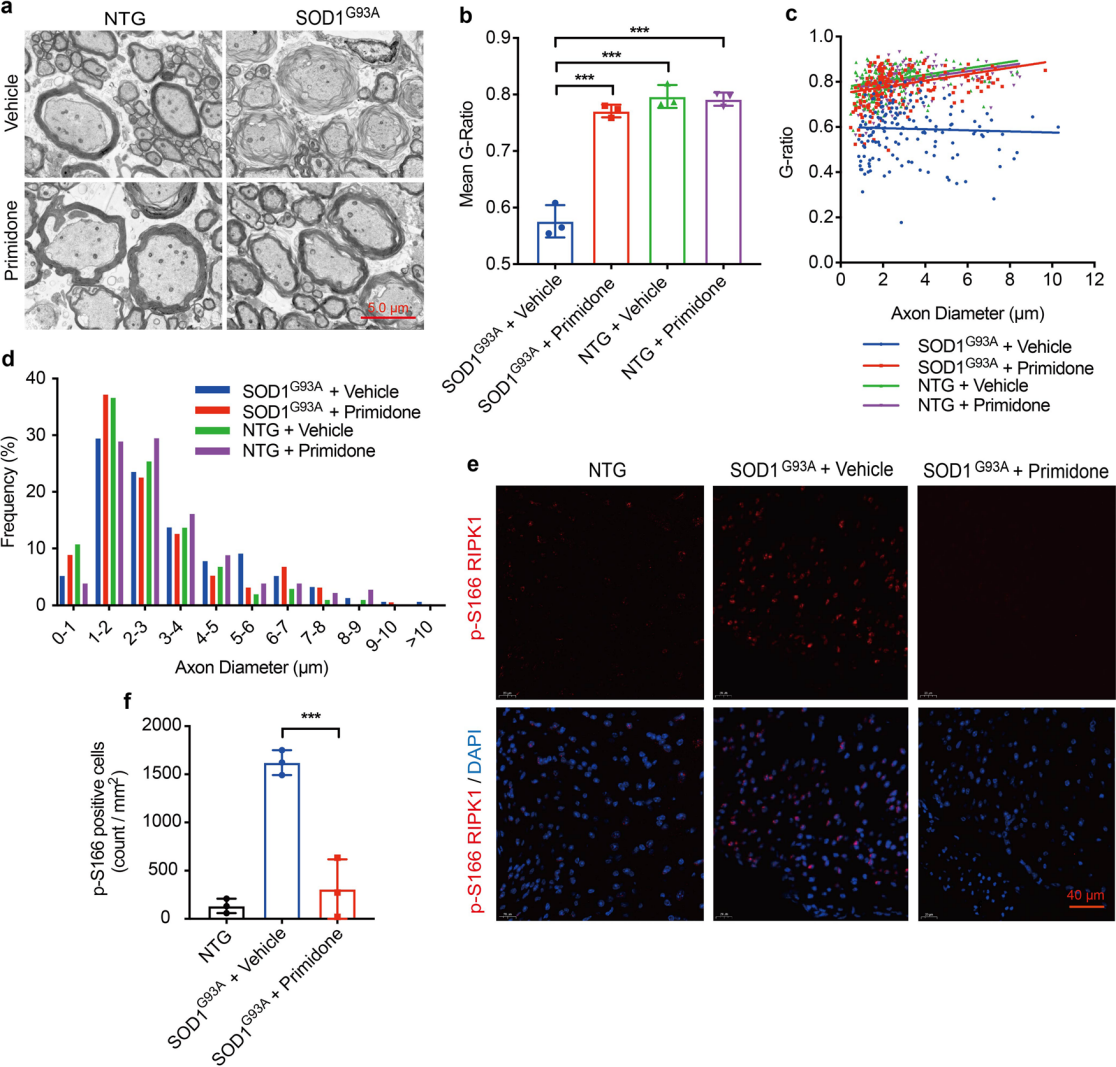
Primidone suppresses RIPK1 activation and ameliorates axonal pathology in the spinal cords of SOD1^G93A^ mice. **a** Electron microscopic images of the motor axon myelination in the lumbar spinal cords of non-transgenic mice (NTG) and SOD1^G93A^ transgenic mice (14 weeks old) with or without primidone treatment. Scale bars, 5.0 μm. The mean g-ratios (**b**), individual g-ratios distribution (**c**) and the distributions of axonal diameters (**d**) in the lumbar spinal cord (L1-L4) of NTG and SOD1^G93A^ transgenic mice with or without primidone treatment. **e** The spinal cord samples were analyzed by immunostaining for p-S166 RIPK1, or DAPI for nuclei (*n* = 3). Scale bars, 40 μm. **f** Cells with activated RIPK1 were quantified by ImageJ. Statistical analysis of g-ratios and cells with activated RIPK1 was conducted with unpaired t-test, Brown-Forsythe and Welch ANOVA test. Data are expressed as mean ± SD, *n* = 3 mice/group, ***P < 0.001

The destruction of myelin sheath is an early pathological finding in SOD1^G93A^ mice, which can contribute to the damage of motor neuron axons.^14^ Therefore, we examined the effect of primidone on myelin sheath around motor neuron axons in the spinal cords of SOD1^G93A^ mice. Similar to that of axonal pathology found in Optn -/- mice,^6^ another ALS model, the axonal pathology of SOD1^G93A^ mice is manifested as a decompaction of myelin sheaths with a decreased g-ratio and an increased number of large-diameter axons, suggesting degeneration and swelling of motor neuron axons (Fig. 2a, b). Treatment with primidone restored the density of myelin sheath around motor neuron axons and the number of axons with large diameters in the spinal cords of SOD1^G93A^ mice (Fig. 2c, d). Using p-S166 RIPK1 immunostaining, a biomarker of activated RIPK1,^8^ we identified a significant increase in the number of cells with activated RIPK1 in the spinal cord of SOD1^G93A^ mice, which was inhibited by primidone treatment (Fig. 2e, f). Thus, these results suggest that inhibition of RIPK1 in SOD1^G93A^ mice with primidone can suppress RIPK1 activation and dysmyelination in the spinal cords of SOD1^G93A^ mice which plays an important role in the onset and progression of axonal dysfunction.

### An ALS clinical biomarker study with off-label use of primidone

The preclinical efficacy of primidone in slowing the disease progession of SOD1^G93A^ mice encouraged us to further investigate the clinical value of primidone in human ALS. Towards this goal, we first conducted a clinical biomarker study to investigate the involvement of RIPK in human ALS patients.

We first tested a very low dose of primidone in a group of ALS patients. The minimum subdividable size is one-eighth of the entire 250 mg/tablet with commercially available primidone. Thus, this off-label 31.25 mg/day primidone use for ALS patients, considerably lower than the original dose listed in instructions as antiepileptic or anti-tremor drug, was expected to be safe. Twenty-nine ALS patients, who were orally administered primidone at the dosage of 31.25 mg every night, completed follow-up visiting back to our ALS center every month during 3 months for measuring the levels of serum RIPK1 and IL-8 by ELISA. The result showed that the serum levels of RIPK1 and IL-8 in the ALS patients treated with primidone at the dosage of 31.25 mg/day were significantly reduced compared with their initial levels at baseline (Supplementary Fig. 3). This pilot study validated the ability of primidone to reduce the serum levels of RIPK1 and IL-8 in ALS patients.

We next doubled the dose of primidone oral treatment to 62.5 mg per day in ALS patients, which is still far lower than the dose recommended in the instruction manual of primidone. One hundred and eighty-nine ALS patients with ages from 30 to 73 years were recruited (Supplementary Table 1). The diagnosis of these ALS patients identified 28 with bulbar onset (B-ALS) and 161 with non-bulbar onset (L-ALS). Of 189 patients enrolled and used at baseline analyses, 176 patients were analyzed for safety of 62.5 mg daily dose of primidone treatment, prescribed off-label with the approval of the Ethics Committee in the First Hospital of Yichang, China. One-hundred-sixty-two of 176 ALS participants completed 24-week follow-up (Supplementary Fig. 4). The demographics of the participants are shown in Supplementary Table 1.

As expected, primidone at this dose of 62.5 mg daily was safe and well tolerated by ALS patients. Although the dizziness and sleepiness as short-term side effects for the first 3 days were noticed in a small percentage (22.16% and 12.50%) of the patients, the incidence of side effects significantly declined over time. Other infrequent side effects included nausea, vomiting and thirsty (Supplementary Fig. 5). None of patients developed any laboratory abnormalities attributable to primidone.

### Primidone improves biochemical parameters in a subset of ALS patients

**Fig. 3:**
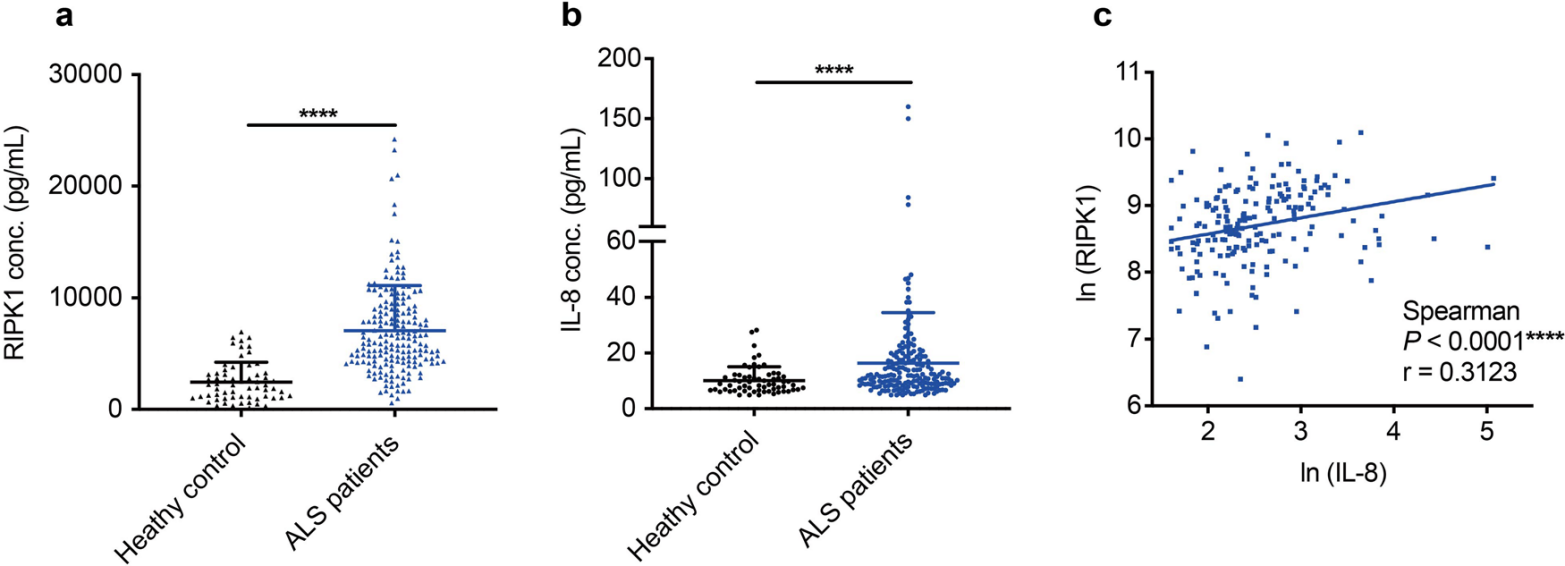
Elevated levels of RIPK1 and IL-8 in circulating serum of ALS patients. **a**, **b** Plotted RIPK1 and IL-8 levels in the serum of individual healthy control (*n* = 63) and baseline of ALS patients (*n* = 189). Graph (**a**, **b**) were plotted in Prism 8 showing mean values with SD. Statistical test used Mann-Whitney test, ****P < 0.0001. **c** Data were Natural Log transformed prior to analysis. Serum RIPK1 levels were positively associated with serum IL-8 levels (*n* = 189) (Spearman correlation coefficient, *r* = 0.3123, with ****P < 0.0001)

The serum levels of RIPK1 have been shown to correlate with the extent of necroptosis in an animal model of non-alcoholic fatty liver disease.^15^ We found that the serum levels of RIPK1 were significantly elevated in ALS patients compared to that of control individuals (Fig. 3a). Necroptosis is a form of necrotic cell death characterized by the early cytoplasmic permeabilization and thus, the serum levels of RIPK1 may be released from necroptotic cells and can be used as a biomarker for necroptosis.^16,17^ Since the activation of necroptosis promotes inflammation, such as increased transcription and release of IL-8,^16^ we also screened for the levels of proinflammatory cytokines. We found that the serum levels of IL-8 and TNF-α were significantly higher in patients than that in healthy controls (Fig. 3b and Supplementary Table 2). Positive associations were found between serum RIPK1 and IL-8 levels of ALS patients in our study (Fig. 3c). These data suggest an abnormally active state of necroptosis and inflammation in ALS patients.

**Fig. 4:**
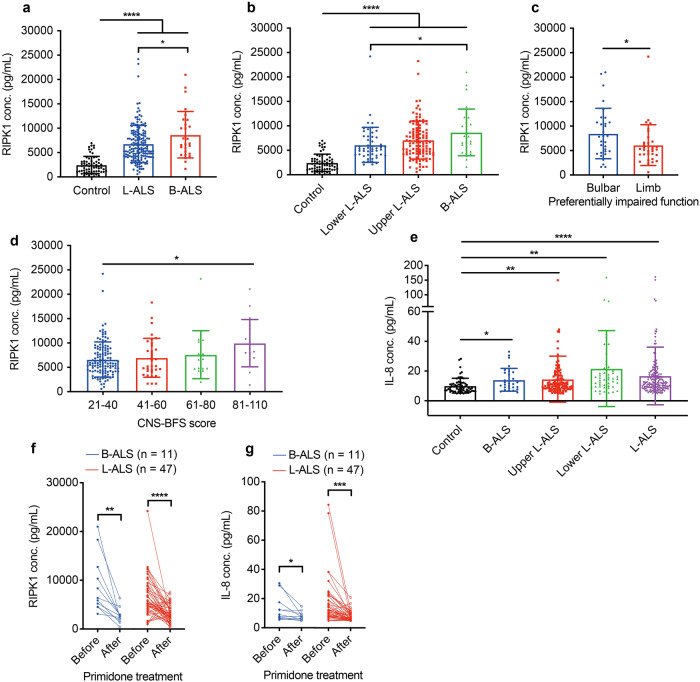
Primidone reduces the elevated serum levels of RIPK1 and IL-8 in ALS. **a** RIPK1 levels in serum of patients with B-ALS (*n* = 28) is higher than that of L-ALS (*n* = 161). *P < 0.05, ****P < 0.0001, unpaired t test. **b** RIPK1 levels in serum of patients with B-ALS (*n* = 28) is higher than that of lower-limb onset ALS (*n* = 50). *P < 0.05, ****P < 0.0001, unpaired t test. **c** RI*P*K1 levels in serum of patients with severe bulbar or limb symptoms, which were defined as ∆bulbar/∆total (*n* = 31) or ∆limb/∆total (*n* = 32) highest sextile. ∆total = 48 − [ALSFRS-R total score], ∆bulbar = 12 − [ALSFRS-R bulbar subscore], ∆limb = 24– [ALSFRS-R limb subscore]. *P < 0.05, Welch’s *t* test. **d** RIPK1 levels in serum of patients with different CNS-BFS scale scores (*n* = 189). *P < 0.05, unpaired *t* test. **e** IL-8 levels in serum of B-ALS (*n* = 28), upper L-ALS (*n* = 111), lower L-ALS (*n* = 50), or L-ALS (*n* = 161) are higher than in serum of healthy control (*n* = 63). There is no significant difference in IL-8 serum levels between different ALS subgroups (*P* > 0.05). *P < 0.05, **P < 0.01, ****P < 0.0001, Welch’s *t* test. **f**, **g** Changes in the serum RIPK1 levels and IL-8 levels before and after 24 weeks of primidone treatment with daily dosage 62.5 mg. Paired *t* test was used for (**f**, **g**). #Bulbar-onset ALS before versus after primidone treatment for 24 weeks, *Limb-onset ALS before versus after primidone treatment for 24 weeks, #*P* < 0.05, ##*P* < 0.01, ***P < 0.001,****P < 0.0001. All data are expressed as mean ± SD

ALS is heterogeneous in sites of disease onset and differently impaired limb, bulbar and respiratory functions. Next, we investigated whether the levels of abnormally increased circulating RIPK1 were associated with characteristics of patients with ALS. We found that the serum RIPK1 value in B-ALS was higher than in L-ALS (*P* < 0.05) (both upper and lower limb onset) (Fig. 4a, b). We further analyzed the association of circulating RIPK1 and symptoms in patients with ALS. Significantly higher circulating RIPK1 levels were found in patients with more severe bulbar symptoms than those with more severe limb symptoms in our study (*P* < 0.05) (Fig. 4c). Moreover, serum RIPK1 levels in patients with ALS are correlated with the severity of bulbar symptoms, as indicated by higher serum RIPK1 levels in patients with more severe bulbar symptoms (Fig. 4d). The IL-8 levels of any ALS subgroup were statistically higher than healthy control, while no significant difference in IL-8 serum levels between the different ALS subgroups was detected (Fig. 4e). These data suggest a preferentially abnormal activation of RIPK1 and necroptosis in ALS with bulbar onset and ALS with more severe bulbar symptoms and also increased IL-8 in peripheral as a biomarker for inflammation in ALS.

To characterize the effects of primidone on necroptosis and inflammation in ALS patients, we analyzed the changes of RIPK1 and IL-8 in ALS patients after primidone treatment. Our study showed that the serum levels of RIPK1 and IL-8 in ALS patients treated with primidone significantly decreased overtime (Fig. 4f, g). These data suggest that primidone can reduce necroptosis and inflammatory response in ALS patients.

Collectively, our data demonstrated that the primidone treatment improved biochemical parameters, regardless of B-ALS or L-ALS.

## Discussion

Activation of RIPK1 is known to promote apoptosis, necroptosis and inflammation.^5^ Our animal model study using SOD^G93A^ transgenic mice demonstrated the ability of primidone to reduce axonal pathology and improve motor performance, suggesting that similar to that of Optn -/- mice,^6^ the pathogenesis of SOD^G93A^ transgenic mice also involves the role of RIPK1 in mediating axonal degeneration and neuroinflammation. The efficacy of primidone in ALS mouse model encouraged us to conduct further studies in human ALS.

Our study demonstrates the increased presence of RIPK1 and IL-8 in the serum of ALS patients, supporting the activation of RIPK1-mediated inflammatory response in human ALS. Furthermore, we demonstrate the increased levels of RIPK1 in serum of ALS with bulbar onset and ALS with severe bulbar symptoms, suggesting the role of RIPK1 and necroptosis in mediating bulbar dysfunction in ALS. RIPK1 is being investigated clinically as an important pharmacological target for the treatment of a variety of human diseases characterized by inflammation and degeneration.^5^ In particular, RIPK1-dependent programmed cell death, including RDA and necroptosis, and inflammation has emerged as potential important mechanisms in promoting the progressive cell death of motor neurons in the pathogenesis of ALS.^6,18,19^ A RIPK1 inhibitor has been advanced into Phase II human clinical trials for the treatment of ALS. Discovery of clinical biomarkers for RIPK1 activation in ALS may help with the design and development of future clinical studies and for patients stratification in interpreting clinical testing results. In addition, our study also suggests the potential value of using primidone, an FDA-approved antiepileptic drug with a 70-year safety track record, for the treatment of ALS, which may be investigated in a double-arm clinical study with controls in future.

Our study also demonstrated a correlation with increased levels of RIPK1 and that of IL-8 in the serum of ALS patients compared to that of healthy control individuals. Activation of necroptosis leads to robust induction of IL-8 expression and secretion.^16^ Elevated levels of IL-8 expression have been found in the monocytes from ALS patients which demonstrated a unique inflammation-related gene expression profile.^20^ Thus, the elevated levels of IL-8 in our study may be of monocytic origin. It is possible that increased peripheral proinflammatory monocytes may enter CNS to directly modulate the neuroinflammatory milieu and course of ALS progression. Alternatively, enhanced peripheral inflammation may indirectly modulate CNS disease progression by affecting the proinflammatory status of T cells, dendritic cells, or natural killer cells in lymph nodes, spleen, or peripheral circulation. The serum RIPK1 levels in B-ALS were higher than in L-ALS and higher circulating RIPK1 levels were found to correlated with the severity of bulbar symptoms, suggesting a preferentially abnormal activation of RIPK1 and necroptosis in ALS with bulbar onset and ALS with more severe bulbar symptoms. The ability of primidone to reduce the levels of serum IL-8 and RIPK1 in ALS patients suggests a possible role of RIPK1 and necroptosis in regulating the expression of IL-8. It will be interesting to investigate the role of RIPK1 in regulating the expression of IL-8 in the CNS in the future. Before the development of a RIPK1-specific PET image probe, we suggest the use of RIPK1 and IL-8 in serum as a biomarker for measuring the activation of RIPK1 in the CNS.

There are several potential limitations to our study that have to be considered. First, longer follow up are needed to assess the long-term safety and effectiveness of primidone treatment in ALS patients. Second, this study has limitations due to its retrospective, non-RCT study design. A RCT study with placebo control, which has higher validation effectiveness than Single-arm research, is being considered for further implementation to observe the therapeutic effects of primidone in ALS.

ALS is a heterogeneous disease involving a variety of pathogenic genes such as OPTN, NEK1, and TBK1 and risk factors.^5,21,22^ Mutant mice with deficiencies in OPTN, NEK1, and TBK1 are sensitized to the activation of RIPK1 kinase and RIPK1-dependent programmed cell death which can be blocked by RIPK1 inhibitor.^6–8,23^ Clinical trials should be conducted to test the efficacy of primidone in ALS patients, particularly the subtypes caused by loss-of-function mutations of OPTN, NEK1, TBK1, and other ALS risk genes. Repurposing primidone may provide a promising therapeutic strategy for this devastating life-threaten disease. In addition, the activation of RIPK1 has also been implicated in mediating a variety of inflammatory diseases.^5,24^ For example, RIPK1 has been implicated in mediating the production of proinflammatory cytokines by interacting with NSP12 in COVID-19.^25^ Primidone has been suggested to inhibit COVID-19-associated cytokine release syndrome (CRS).^10^ Thus, the effect of primidone on the treatment of other inflammatory diseases may also be considered.

In conclusion, we conducted a repurpositional biomarker study using an old drug, primidone which has been shown to also inhibit RIPK1,^10^ with animal ALS model and human ALS participants. Treatment with primidone delayed the onset and progression of motor deficits, histological pathology and body-weight loss in SOD^G93A^ mice, validating the effect of primidone to inhibit RIPK1 and rescue motor functions in vivo. In human ALS participants recruited in our study, we found that dosing with primidone reduced the serum levels of RIPK1 and IL-8, which were significantly higher in ALS patients than that of healthy individuals. Serum RIPK1 levels in patients with ALS are positively correlated with the severity of bulbar symptoms. These data demonstrate the value of RIPK1 and IL-8 in serum as biomarkers in clinical studies developing RIPK1 inhibitor for the treatment of ALS. Repurposing primidone may provide a promising therapeutic strategy for ALS. The effect of primidone on the treatment of other inflammatory diseases may also be considered.

## Materials and methods

### Ethics statement

All the animal experiments were performed in accordance with the Guide for the Care and Use of Laboratory Animals and were approved by the Animal Care Committee of Three Gorges University, China (2019060B). The approval of primidone off-label prescription for ALS patients was obtained from the Ethics Committee in the First Hospital of Yichang, affiliated to China Three Gorges University (ChiCTR2200060149).

### Reagents and antibodies

We used the following reagents: primidone (Wuhan Xinxinjiali Bio-Tech Co., Ltd, 282098-10g); anti-pS166-RIPK1 for IF (BioLynx Technology, BX60008).

### Transgenic mice

TB6.Cg-Tg (SOD1^G93A^) 1Gur/J and non-transgenic C57BL6J mice (NTG) were obtained from the Jackson Laboratory. Mice were housed in a SPF facility with a 12:12-h light/dark cycle and controlled temperature of 22 ± 2 °C. All the animal experiments were performed in accordance with the Guide for the Care and Use of Laboratory Animals and were approved by the Animal Care Committee of Three Gorges University, China (2019060B).

### Primidone treatment in SOD1^G93A^ mice

Animals were grouped by simple randomization using a random number table. SOD1^G93A^ mice were administered with primidone at a dose of 25 mg/kg via gavage once a day from 10 weeks after birth. The vehicle group of mice was treated with 1% CMC-Na via gavage. Efficacy was measured using endpoints indicative of neuro-protective function, including open-field, body weight and muscle weakness. Body weights were recorded once a week. Above other measurements were performed twice a week from 10 weeks of age to the end stage. The open field was a square box with black walls and a light-colored floor. Mice were placed in the center of the open Field and allowed to explore for 3 min. Computer-aided video tracking system (DIGBEHV, Shanghai Jiliang) was used to track the time and distance. The total distance traveled in the open field was used as a measure of motor performance. Neurological scoring that indicates muscle weakness of SOD1^G93A^ mice followed the guideline provided by the Jackson Laboratory.^26^ Detailed rules for evaluating the neurological score of 0 to 4 was described in the guideline. When the score is more than 2, it is considered as the time of onset.^26^

### Transmission electron microscopy

SOD1^G93A^ and control mice were sacrificed 14 weeks of age and perfused with 4% paraformaldehyde followed by fixation solution of 2.5% glutaraldehyde in 0.1 M sodium cacodylate buffer (pH 7.4). Small pieces (4 mm^3^ cubes) of lumbar spinal cords (L1-L4) from a perfusion-fixed animal were post-fixed for at least 2 h at RT in the above fixative. The samples were washed three times in 0.1 M cacodylate buffer for 15 min and fixed with 1% osmium tetroxide (OsO_4_)/1.5% potassium ferrocyanide (KFeCN_6_) for 2 h, washed three times in 0.1 M cacodylate buffer for 15 min, then alcohol-grade dehydration. The samples were then put in propylene oxide for 1 h and infiltrated overnight in a 1:1 mixture of propyleneoxide and TAAB Epon (Marivac Canada St. Laurent, Canada). The samples were then embedded in TAAB Epon and polymerized at 60 °C for 48 h. Ultrathin sections (60 nm) were cut with a Leica EM UC7 microtome, examined using a Hitachi HT7700 Transmission electron microscope and images were recorded with an AMT 2k CCD camera.

### Axonal diameter and g-ratio determination

Axon diameters on the images recorded by transmission EM were measured using ImageJ. G ratio is defined as the ratio of the inside diameter to the outside diameter of each axon fiber.^6,27^ At least 150 axons were measured in the ventral white matter of the lumbar spinal cord (L1-L4) of 3 14-week-old mice in each analysis.

### Immunofluorescence

Animals were sacrificed and perfused with 4% paraformaldehyde. The lumbar spinal cords (L1–L4) were removed and post-fixed for at least 2 h at RT in 4% paraformaldehyde. The paraffin wax embedded tissue samples sliced into 4 μm thick sections. After blocking with 3% BSA for 45 min, the slides were incubated with anti-pS166-RIPK1 at 4 °C overnight, after washing with PBS, the slides were incubated with secondary antibodies (CY-3, or anti-rabbit, Jackson Immunoresearch; 1:250) for 50 min. The sections were then rinsed in PBS and dropped DAPI for 10 min. Imaging was performed using a NIKON Biological fluorescence microscope and NIS Elements software.

### Off-label primidone approvals and patient consent

The approval of primidone off-label prescription for ALS patients was obtained from Ethics Committee in the First Hospital of Yichang, affiliated to China Three Gorges University (ChiCTR2200060149). All participating ALS patients provided their written informed consent to treatment with primidone “off-label” medication, for research review and analysis of medical records.

### Participant selection criteria and off-label primidone medication protocol

Eligible patients aged 18–80 years had a diagnosis of possible, probable laboratory-supported, probable, or definite ALS by EI Escorial Criteria.^28^ The reasons for exclusion of potential participants included pregnancy; exposure to immunosuppressive therapy within 4 weeks of screening; active autoimmune disease or infection (including hepatitis B, hepatitis C, or HIV), unstable psychiatric, dementia; or clinically significant abnormal safety laboratory values.

Control serum samples were obtained from healthy volunteers over 18 years of age, without any history or clinical signs of diseases implicated to involve necroptosis, such as multiple sclerosis, Alzheimer’s disease, and Parkinson’s disease, inflammatory bowel disease, sepsis, viral infection, hepatitis, neuronal and renal degeneration, tumor metastasis, ischemia–reperfusion-induced tissue injury, stroke, myocardial infarction, renal failure, systemic inflammatory response syndrome and other inflammation, infection, autoimmune and degenerative diseases.^19,29,30^

The oral dose of primidone tablets (250 mg/tablet, CHENPON, China) was one-eighth tablet per night for the first 3 days, after which the dose was increased to one-fourth tablets per day in two divided doses with a target dose of 62.5 mg daily.

### Neurological function assessment in patients with ALS

The comprehensive neurological function of respiration, bulbar and limbs for patients with ALS was assessed by ALS Functional Rating Scale Revised (ALSFRS-R)^31^ and Center for Neurologic Studies Bulbar Function Scale (CNS-BFS).^32^ Progression rate of ALSFRS-R score (or bulbarsubscore) = ([normal score] − [score at the time])/[duration of disease]. Progression rate of CNS-BFS score = ([score at the time] − [normal score])/[duration of disease].

### Serological assays

Serum levels of RIPK1 of ALS patients and control subjects were determined by an ELISA kit (catalog SEE640Hu; Cloud-Clone Corp, China) according to the manufacturer’s instructions. Serum levels of the cytokines TNF-α, IL-1β, IL-2R, IL-6, IL-8, and IL-10 were determined by the automated chemiluminescent immunoassay system IMMULITE 1000 (Siemens Healthcare Diagnostics Inc, USA).

### Statistics

Data are expressed as the mean value ± standard deviation. Continuous variables were tested for distribution normality using the Shapiro-Wilk test first. Categorical variables are expressed as counts and percentages. Independent t-tests or Mann-Whitney tests were applied to continuous variables. The chi-square tests or Fisher’s exact tests were used for categorical variables. Figures were generated using GraphPad Prism Version 8.0. A two-tailed *P* < 0.05 was considered to be statistically significant.

### Supplementary information

Supplementary materials

## Data Availability

Reasonable requests for raw and analyzed data and materials are available from the corresponding author and will be reviewed by the Ethics Committee in the First Hospital of Yichang. Patient data might be subject to patient confidentiality.
