# Supplementary material for: Elevated peripheral levels of receptor-interacting protein kinase 1 (RIPK1) and IL-8 as biomarkers of human amyotrophic lateral sclerosis
Source: Signal Transduct Target Ther. 2023 Dec 13;8:451. doi: 10.1038/s41392-023-01713-z (PMC10716192; doi:10.1038/s41392-023-01713-z)
Supplement: Supplementary file 1 — Supplementary materials [file 41392_2023_1713_MOESM1_ESM.docx]

Supplementary Materials for

Elevated peripheral levels of receptor-interacting protein kinase 1 (RIPK1) and IL-8 as biomarkers of human amyotrophic lateral sclerosis

Jun Wei^#^, Min Li^#^, Zhi Ye^#^, Xinqian Hu, Xiaoyan He, Jia Wang, Gaofeng Chen, Chengyu Zou, Daichao Xu, Hongbing Zhang, Junying Yuan* and Yunhong Zha*.

Correspondence to: Junying Yuan*, Junying_yuan@sioc.ac.cn; Yunhong Zha*, yzha7808@ctgu.edu.cn.

**This PDF file includes:**

Methods

Figures. S1 to S5

Tables S1 to S2

Methods

In vitro kinase assay

HEK293T cells were transfected with an expression vector for Flag-mRIPK1 Full Length and treated with Nec-1 for 14 hours to prevent autophosphorylation of RIPK1. These cells were lysed by 1% NP-40 buffer without NaF/Na3VO4. Flag-mRIPK1 was immunoprecipitated by using ANTI-FLAG® M2 Affinity Gel and washed 2 times with high salt lysis buffer (1% Nonidet P-40, 50 mM Tris-base (pH 7.5), 500 mM NaCl), and then washed 3 times with 1X kinase buffer (10 mM MgCl_2_, 20 mM HEPEs, pH 7.5). Flag-RIPK1 beads was incubated with the solution including different combinations of 20 mM ATP and different concentrations of primidone at 37 ℃ for 30 min, then eluted by the addition of loading buffer in 95 ℃ for 5 min.


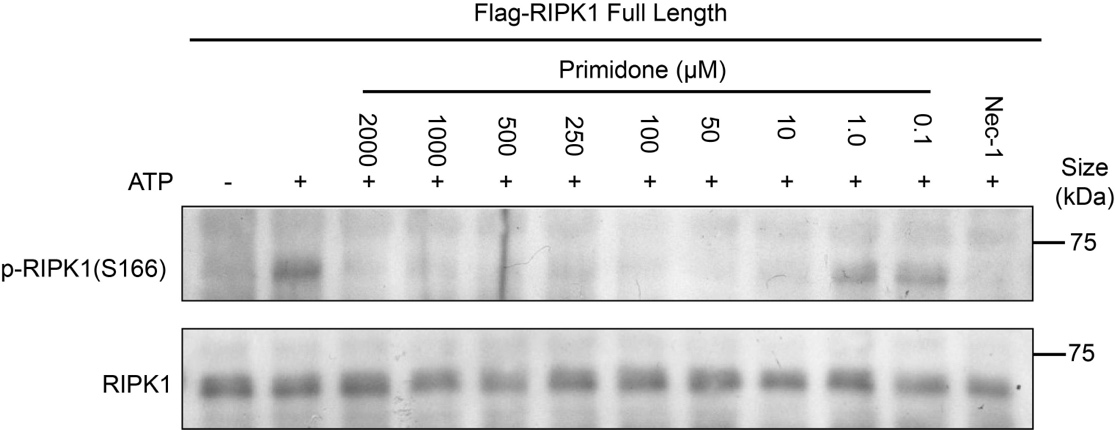


Figure. S1.

**Primidone is a direct inhibitor of RIPK1 kinase activity.** Full length Flag-RIPK1 expressed in 293T cells was immunoprecipitated using anti-Flag beads and incubated with or without 20 mM ATP or with indicated different concentrations of primidone at 37 ℃ for 30 min. The samples were analyzed by western blotting with p-RIPK1 (pS166). 20 μM Nec-1 was used as a positive control.

Full unedited gel for figure. S1


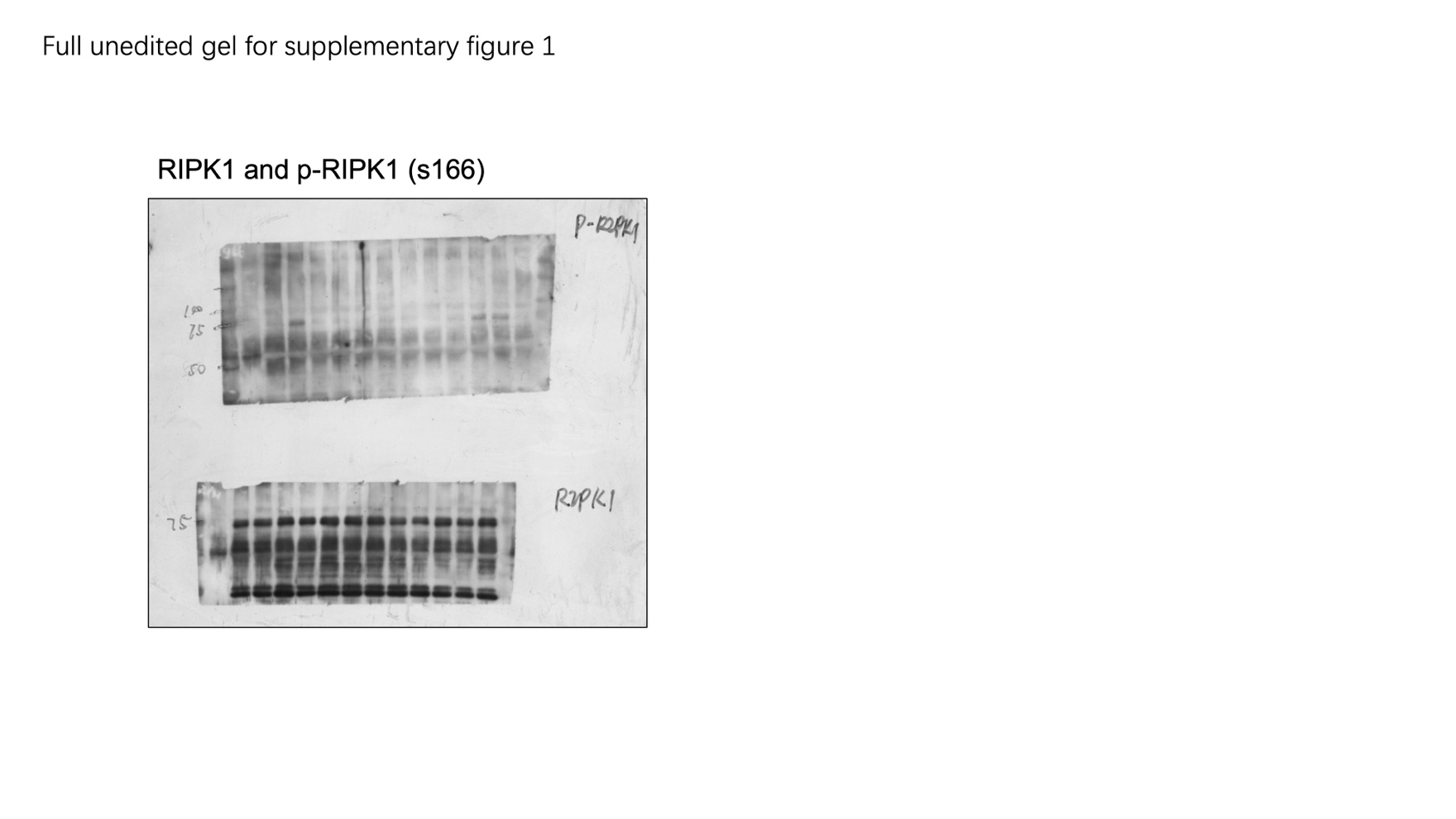


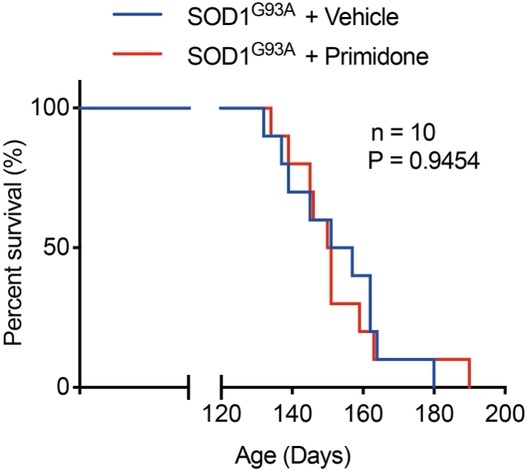


Figure. S2.

**Kaplan–Meier survival plot of SOD1^G93A^ mice.** Statistical analysis of mouse survival was conducted with Log-rank (Mantel-Cox) test, P > 0.05, n = 10 mice/group. Type or paste caption here. Create a page break and paste in the Figure above the caption.


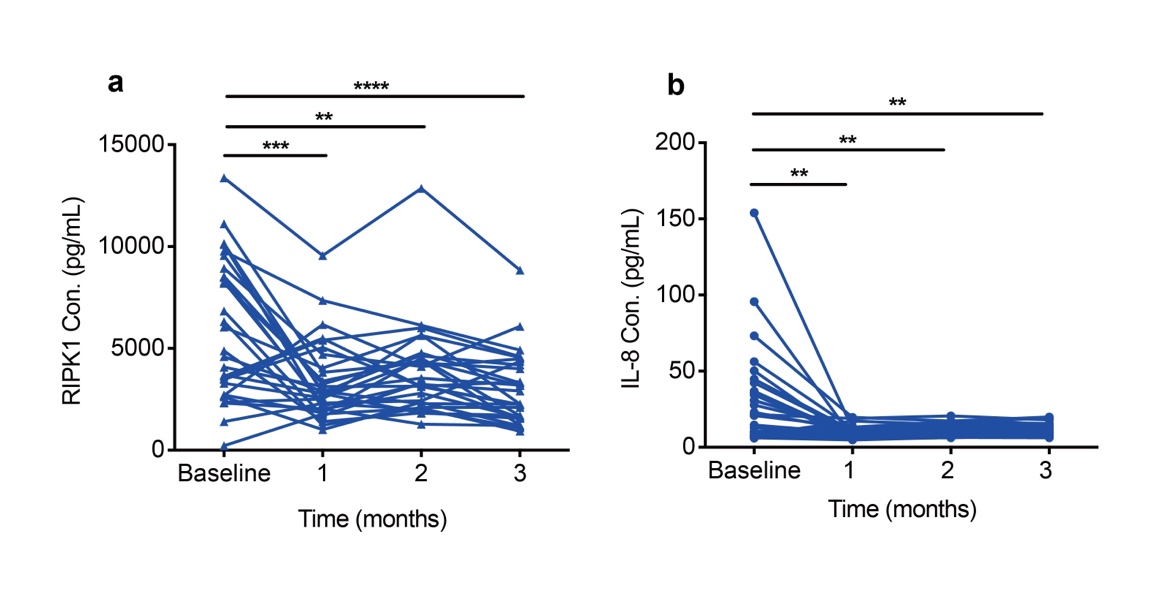


Figure. S3.

**Primidone significantly reduces the abnormally increased serum levels of RIPK1 and IL-8 in ALS patients.** Serum levels of RIPK1 (a) and IL-8 (b) in ALS patients taking primidone at 31.25 mg/day for 3 months were reduced from baseline. N = 29 (15 male and 14 female patients). The mean age was 56.83 years (SD, 10.43 years). The mean onset age was 52.21 years (SD, 11.14 years). Statistical test used paired t test, ***P* < 0.01, ****P* < 0.001, *****P* < 0.0001.


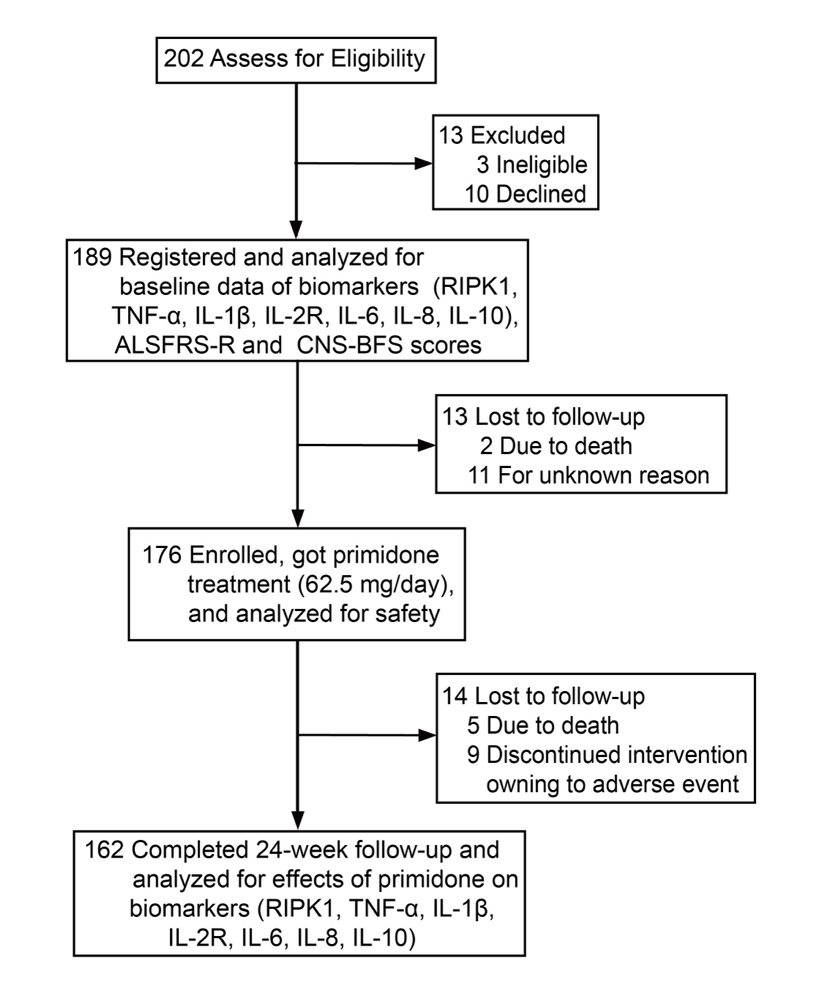


Figure. S4.

Flowchart of the study methodology.


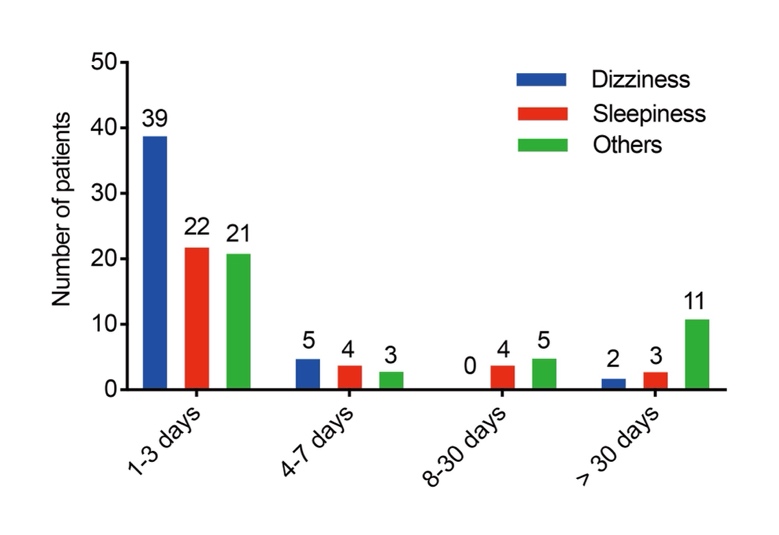


Figure. S5.

Safety of primidone for ALS patients. The most common adverse effects of primidone during the first 3 days were dizziness and sleepiness. The frequencies of primidone side effects significantly declined over time. Other infrequent complaints include nausea, vomiting and thirsty.

Table S1.

**Demographics and characteristics.**

| **Variable** | **B-ALS**  n = 28 | **L-ALS**  n = 161 | **ALS, All**  n = 189 | **ALS, 6-M Completed**  n = 162 | **Healthy Control**  n = 63 | ***P*-Value** |
| --- | --- | --- | --- | --- | --- | --- |
| **Male/Female (N)** | 14/14 | 109/52 | 123/66 | 103/59 | 41/21 | **P* = 0.879^a^  #*P* = 0.086^a^ |
| **Age (years)** | 49.32 (8.80),  31-68 | 50.32 (9.21),  30-73 | 50.17 (9.16),  30-73 | 50.06 (9.02),  30-73 | 52.43 (8.72),  31-78 | **P* = 0.089^b^  #*P* = 0.596^b^ |
| **Onset age (years)** | 47.14 (8.65),  29-67 | 47.99 (9.48),  26-71 | 47.86 (9.37),  26-71 | 47.65 (9.19),  26-71 | - | #*P* = 0.662^b^ |
| **Disease duration (month)** | 25.79 (13.25),  7-62 | 27.55 (16.31),  2-177 | 27.29 (15.91),  2-177 | 28.40 (16.34),  7-117 | - | #*P* = 0.728^c^ |
| **ALSFRS-R score** | 30.67 (8.92),  10-46 | 30.03 (8.33),  8-47 | 30.12 (8.42),  8-47 | 30.52 (8.40),  8-47 | - | #*P* = 0.709^b^ |
| **CNS-BFS score** | 63.07 (21.93), 27-108 | 36.42 (19.33), 21-110 | 40.37 (21.89), 21-110 | 40.12 (22.16),  21-110 | - | #*P* < 0.0001^b^ |
| **ΔALSFRS-R/disease duration** | 0.83 (0.55),  0.04-2.2 | 0.81 (0.50),  0.03-2.56 | 0.81 (0.50),  0.03-2.56 | 0.76 (0.47),  0.03-2.25 | - | #*P* = 0.903^c^ |

*ALS (All) versus Healthy Control, #B-ALS versus L-ALS. a Fisher’s exact test; b Unpaired t test; c Mann-Whitney test. B-ALS, Bulbar-onset ALS; L-ALS, Limb-onset ALS; ALSFRS-R, Amyotrophic Lateral Sclerosis Functional Rating Scale-Revised; CNS-BFS, Center for Neurologic Study Bulbar Function Scale.

Table S2.

**Serum levels of inflammatory factors in ALS patients compared to normal control.**

| **Cytokine** | **Control** | | **ALS patient baseline** | | ***P* value** |
| --- | --- | --- | --- | --- | --- |
|  | **AVG** | **SD** | **AVG** | **SD** |  |
| **TNF-α (pg/mL)** | 5.91 | 1.79 | 6.74 | 2.10 | 0.0053 (**) |
| **IL-2R (U/mL)** | 348.78 | 108.60 | 339.23 | 109.34 | 0.5497 |
| **IL-6 (pg/mL)** | 3.16 | 1.16 | 3.37 | 2.30 | 0.9277 |
| **IL-8 (pg/mL)** | 10.16 | 4.89 | 16.38 | 18.11 | <0.0001 (****) |
| **IL-1β (pg/mL)** | < 5 | - | < 5 | - | - |
| **IL-10 (pg/mL)** | < 5 | - | < 5 | - | - |
| AVG: Average, SD: Standard deviation. ** *P* < 0.01, **** *P* < 0.0001 | | | | | |
